# Prognostic significance of low TSH concentration in patients with COVID-19 presenting with non-thyroidal illness syndrome

**DOI:** 10.1186/s12902-021-00766-x

**Published:** 2021-05-27

**Authors:** Jing Gong, Ding-kun Wang, Hui Dong, Qing-song Xia, Zhao-yi Huang, Yan Zhao, Xing Chen, Fen Yuan, Jing-bin Li, Fu-er Lu

**Affiliations:** 1 Department of Integrated Traditional Chinese and Western Medicine, Tongji Hospital, Tongji Medical College, Huazhong University of Science and Technology, Wuhan, China; 2 Institute of Integrated Traditional Chinese and Western Medicine, Tongji Hospital, Tongji Medical College, Huazhong University of Science and Technology, Wuhan, China; 3 Department of Clinical Laboratory, Tongji Hospital, Tongji Medical College, Huazhong University of Science and Technology, Wuhan, China; 4 China Clinical Research Center, Tongji Hospital, Tongji Medical College, Huazhong University of Science and Technology, Wuhan, China

**Keywords:** COVID-19, Thyroid, Non-thyroidal illness syndrome, Mortality

## Abstract

**Background:**

Low free triiodothyronine (FT3) levels are related to a poor prognosis deterioration in patients with COVID-19 presenting with non-thyroidal illness syndrome (NTI). This study was designed to explore whether free thyroxin (FT4) or thyroid stimulating hormone (TSH) levels affected the mortality of patients with COVID-19 presenting with NTI.

**Methods:**

Patients with COVID-19 complicated with NTI who were treated at our hospital were included in this retrospective study. Patients were divided into low TSH and normal TSH groups, as well as low and normal-high FT4 group, according to the reference range of TSH or FT4 levels. The 90-day mortality and critical illness rates were compared among patients with low and normal TSH levels, as well as among patients with low FT4 levels and normal-high FT4 levels; in addition, differences in demographic and laboratory data were compared. A Kaplan-Meier analysis and Cox proportional hazards models were used to assess the associations of TSH and FT4 levels with mortality.

**Results:**

One hundred fifty patients with low FT3 levels and without a history of thyroid disease were included, 68% of whom had normal FT4 and TSH levels. Critical illness rates (74.07% VS 37.40%, *P* = 0.001) and mortality rates (51.85% VS 22.76%, *P* = 0.002) were significantly higher in the low TSH group than in the normal TSH group. Although no significant difference in the critical illness rate was found (*P* = 0.296), the mortality rate was significantly higher in the low FT4 group (*P* = 0.038). Low TSH levels were independently related to 90-day mortality (hazard ratio = 2.78, 95% CI:1.42–5.552, *P* = 0.003).

**Conclusions:**

Low FT4 and TSH concentrations were associated with mortality in patients with COVID-19 presenting with NTI; moreover, low TSH levels were an independent risk factor for mortality in these patients.

## Background

The outbreak of COVID-19 has posed a serious health threat globally. It plays an important role in disease prevention and control to elucidate the clinical features of COVID-19, identify prognostic predictors and explore potential treatment methods. A study from China found that approximately 27.52% of patients with COVID-19 had non-thyroidal illness syndrome (NTI) [1], which is characterized by a decrease in triiodothyronine (T3) levels and/or a decrease in thyroxin (T4) levels without an increase in thyroid-stimulating hormone (TSH) levels [2, 3]. NTI typically occurs in patients admitted to intensive care units (ICUs) and is closely associated with the disease condition and prognosis [3, 4]. The above-mentioned study found that patients with NTI had up to 2.5-fold increased ratio for severe COVID-19 [1].

Low free T3 (FT3) levels in patients with NTI are typically related to a worse prognosis [5]. For example, low T3 syndrome was found to be a strong prognostic predictor of death in patients with cardiac disorders [6]. In addition to FT3 levels, free T4 (FT4) or TSH levels might affect the mortality of patients with NTI [7]. A study reported a worse prognosis for patients with sepsis and NTI presenting low T3 and T4 levels compared with those with low T3 levels alone [8]. According to another study from Turkey, low FT3 levels and increased FT4 levels are considered independent predictors of long-term mortality risks in chronic patients with NTI [9]. Clinically, patients with COVID-19 presenting with low FT3 and TSH levels appear to have a higher mortality rate, which is not completely consistent with previous NTI studies. Researchers have not clearly determined whether FT4 and TSH levels associated with NTI are related to the prognosis of patients with COVID-19. The clinical characteristics of NTI in patients with COVID-19 need further elaboration. This study retrospectively analysed the clinical characteristics of NTI and explored whether TSH or FT4 levels were independent risk factors for mortality of hospitalized patients with both COVID-19 and NTI.

## Methods

### Patients

This retrospective cohort study was conducted according to STROBE guidelines. Data were collected from hospitalized patients who were admitted to Tongji Hospital between January and March 2020. By searching electronic medical records, data, including patient age, sex, medical history, disease onset time, discharge or death time, thyroid function, blood cell analysis and other inflammatory parameters, were included in the analysis. Data collection was performed before 20 April, and the data used were anonymized before use. The inclusion criteria were as follows: 1) patients with a confirmed COVID-19 diagnosis who underwent thyroid function examinations, 2) no previous thyroid disease, and 3) FT3 levels below the reference range without TSH levels above the reference range. The reference range for FT3 levels is 3.1–6.8 pmol/L, the reference range for FT4 levels is 12–22 pmol/L, and the reference range for TSH levels is 0.27–4.2 μIU/mL. The reference range of thyroid function in the manual of Roche manufacturers was adopted according to after verification in healthy subjects.

### Endpoints

The primary endpoints were 90-day death events and critical illness events. According to the disease severity guidelines formulated by the National Health Commission of China, namely, the “New Coronavirus-Infected Pneumonia” Severe and Critical Diagnosis and Treatment Program (Second trial version, Number 2020–127) [10], patients were classified as mild, severe and critical cases. Severe cases were defined as a blood oxygen saturation less than 93% or respiratory frequency greater than 30 in the resting state; critical cases were designated as patients who required mechanical ventilation.

### Examination method

Examinations were conducted by the clinical laboratory of Tongji Hospital. Blood FT3, FT4 and TSH levels were tested using a Roche electrochemiluminescence method. Blood cells, including white blood cells (WBC) and leukomonocytes (LC), were detected using fluorescence staining and flow cytometry. Ultrasensitive C-reactive protein (CRP) levels were measured using immunoturbidimetry. Procalcitonin (PCT) levels were determined using a Roche electrochemiluminescence assay, and ferroprotein levels were measured using a Roche granule-enhanced immunoturbidimetry, Siemens chemiluminescence or Roche electrochemiluminescence method. Alanine aminotransferase (ALT) and aspartate aminotransferase (AST) levels were determined using the Roche kinetic method. Lactate dehydrogenase (LDH) levels were detected using a Roche colorimetric assay, and cardiac troponin I (cTnI) levels were measured using a Roche chemiluminescence microparticle immunoassay. Creatinine (Cr) and urea nitrogen (UREA) levels were analysed using a Roche urease assay. D dimer (D2D) levels were detected using STAGO immunoturbidimetry. Clinical and laboratory data were included at the time point of the thyroid function examination. If patients had more than one thyroid test during their hospitalization, only the first result was included in the analysis.

### Statistical analysis

For descriptive variables, continuous data are presented as the medians and interquartile ranges (IQRs), and categorical data are presented as counts and percentages. A *P* value less than 0.05 indicated a significant difference. The chi-square test was performed to compare the proportions of critical illness and death among patients with different FT4 levels or with different TSH levels; Fisher’s exact test was used for a frequency < 5. According to the reference range, patients were divided into low TSH (< 0.27 μIU/mL) and normal TSH (0.27–4.2 μIU/mL) groups or low FT4 (< 12 pmol/L) and normal-high FT4 (≥12 pmol/L) groups. The Mann-Whitney test was used to compare the differences in blood cell counts, history of encephalopathy, levels of inflammatory factors and other biochemical parameters between groups. The Kaplan-Meier analysis and log-rank test were employed to assess the probability of cumulative 90-day mortality. Cox proportional hazards models were used to explore whether TSH levels were associated with the prognosis. Analyses were performed using SPSS 20.0 software.

## Results

### Clinical features of the included patients

Nine hundred forty-five patients with COVID-19 received thyroid function examinations. Among them, 150 patients had no history of thyroid disease and had FT3 levels < 3.1 pmol/L and TSH levels ≤4.2 μIU/mL. Of the 150 included patients, 69 were female and 81 were male, and the median age was 69.5 years (IQR: 61–79 years; Range: 32–92 years). Regarding disease severity, 66 patients (44%) were critically ill, 59 patients (39.33%) were seriously ill, 25 patients (16.67%) were mildly ill, and 46 patients (30.67%) died. None of the patients had a history of pituitary gland disease.

### Profiles of FT4 and TSH levels among included patients with low FT3 levels

As shown in Table 1, among patients with low serum FT3 levels, 68% had normal FT4 and TSH levels. Additionally, 14% had normal FT4 and decreased TSH levels. The proportion of patients with decreased FT4 levels and normal TSH levels was 9.33%. Approximately 4.67% of patients had elevated FT4 and normal TSH levels. Few patients had reduced TSH and abnormal FT4 levels.

**Table 1:** Numbers and ratio of different FT4 and TSH levels in patients with COVID-19 and low FT3 levels

		FT4		
		> 22 pmol/L	12–22 pmol/L	< 12 pmol/L
**TSH**	**0.27–4.2** uIU/mL	**7 (4.67%)**	**102 (68%)**	**14 (9.33%)**
	**< 0.27** uIU/mL	**4 (2.67%)**	**21 (14%)**	**2 (1.33%)**

*FT3* free triiodothyronine, *FT4* free thyroxine, *TSH* thyroid stimulating hormone

### Comparison between the low TSH and normal TSH groups

The patients were divided into a low TSH group and a normal TSH group according to whether the TSH level was within the reference range. Regarding baseline features, no significant differences were observed in age or sex between the low TSH and normal TSH groups. As shown in Table 2, critical illness and mortality rates were significantly higher in the low TSH group than in the normal TSH group.

**Table 2:** Comparison between low TSH and normal TSH groups in patients with COVID-19 presenting with NTI

	Overall (*n* = 150)	Low TSH (*n* = 27)	Normal TSH (*n* = 123)	*P* value
Age, y, M (IQR)	69.5 (61,79)	68 (62.5, 78)	70 (60.5, 79)	0.924
Gender (female n, %)	69 (46%)	12 (44.44%)	57 (46.34%)	0.858
Critical illness (n, %)	66 (44%)	20 (74.07%)	46 (37.40%)	0.001
Mortality (n, %)	42 (28%)	14 (51.85%)	28 (22.76%)	0.002
History of cerebropathy (n, %)	17 (11.33%)	4 (14.81%)	13 (10.57%)	0.551
WBC, *10^9/L, M (IQR)	10.73 (7.795,16.795)	14.08 (10.46, 21.2)	10.2 (7.19, 16.37)	0.023
LC, *10^9/L, M (IQR)	1.45 (0.945,1.87)	1.13 (0.82, 1.97)	1.45 (0.985, 1.87)	0.572
CRP, mg/L, M (IQR)	91.2 (41.825,177.275)	162.6 (70.9, 224.9)	84 (36.45, 155.7)	0.013
Ferroprotein, ug/L, M (IQR)	1314.7 (631.92395.6)	1879.35 (1042.83, 2861.8)	1166.2 (562.35, 2147.15)	0.047
PCT, ng/mL, M (IQR)	0.19 (0.08,0.99)	0.39 (0.115, 4.16)	0.17 (0.08, 0.86)	0.084
ALT, U/L, M (IQR)	44.5 (22.25,73.75)	57 (29, 69.5)	42 (21, 76)	0.219)
AST, U/L, M (IQR)	44.5 (26,75.75)	57 (37.5, 109.5)	42 (25, 69.5)	0.042
ALB, g/L, M (IQR)	35.05 (30.175,39.15)	36.3 (33.5, 39.3)	34.9 (30.15, 38.8)	0.605
LDH, U/L, M (IQR)	221 (180,282)	279 (188, 413)	216.5 (174, 271.5)	0.038
CR, umol/L, M (IQR)	87 (68.25,117.75)	88 (76, 184)	87 (67, 114.5)	0.194
UREA, mmol/L, M (IQR)	8.9 (5.7,15.85)	14.1 (9.7, 26.35)	7.6 (5.2, 14.2)	< 0.001
cTnI, pg/mL, M (IQR)	18.05 (6.35,197.875)	70.7 (11.2, 755.65)	13.8 (6.2, 162.7)	0.098
D2D, ug/mL FEU, M (IQR)	5.365 (1.46,19.045)	8.41 (2.47, 17.74)	4.5 (1.39, 19.06)	0.316
FT3, pmol/L, M (IQR)	2.665 (2.303,2.888)	2.68 (2.35, 2.92)	2.66 (2.31, 2.88)	0.696
FT4, pmol/L, M (IQR)	16.05 (13.623,18.345)	16.39 (13.53, 18.89)	16 (13.75, 18.25)	0.687
TSH, uIU/mL, M (IQR)	0.96 (0.412,1.848)	0.16 (0.10, 0.21)	1.29 (0.69, 2.03)	/

*NTI* non-thyroidal illness syndrome, *M (IQR)* Median (interquartile range), *WBC* white blood cell, *LC* leukomonocyte, *CRP* ultrasensitive C-reactive protein, *PCT* procalcitonin, *ALT* alanine aminotransferase, *AST* aspertate aminotransferase, *ALB* albumin, *LDH* lactate dehydrogenase, Cr Creatinine, *UREA* urea nitrogen, *cTnI* cardiac troponin I, *D2D* D dimmers

To explore the possible causes or relevant factors of decreased TSH, the history of encephalopathy, biochemical markers (ALT, AST, ALB, LDH, Cr, UREA and cTnI), blood cell (WBC and LC) counts, inflammatory parameters (CRP, ferroprotein and PCT), and D dimer (D2D) levels were compared between the low and normal TSH groups. As shown in Table 2, significant differences between the two groups were observed in inflammatory parameters, including WBC counts (*P* = 0.023), CRP levels (*P* = 0.013) and ferroprotein levels (*P* = 0.047). In addition, significant differences in AST (*P* = 0.042), LDH (*P* = 0.038), and UREA levels (*P* < 0.001) were also detected. However, no significant differences were found in the history of encephalopathy (*P* = 0.551), LC (*P* = 0.572), PCT (*P* = 0.084), D2D (*P* = 0.316), ALT (*P* = 0.219), ALB (*P* = 0.605), Cr (*P* = 0.194) or cTnI (*P* = 0.098) levels. In addition, no significant difference was found in FT3 and FT4 levels between the low TSH group and the normal TSH group.

### Comparison between the low FT4 and normal-high FT4 groups

The patients were also divided into a low FT4 group and a normal-high FT4 group according to whether the FT4 level was below the reference range. No significant differences were observed between the two groups in age and gender (Table 3). Although a significant difference in the critical illness rate was not identified, the mortality rate was significantly higher in the low FT4 group (*P* = 0.038).

**Table 3:** Comparison between low and normal-high FT4 groups in patients with COVID-19 presenting with NTI

	Low FT4 (*n* = 16)	Normal-high FT4 (*n* = 134)	*P* value
Age, y, M (IQR)	64.5 (41.75, 71.75)	70 (62.25, 79.75)	0.077
Gender (female n, %)	8 (50%)	61 (45.52%)	0.734
Critical illness (n, %)	9 (56.25%)	57 (42.54%)	0.296
Mortality (n, %)	8 (50%)	34 (25.37%)	0.038
WBC, *10^9/L, M (IQR)	10.35 (8.34, 26.87)	10.92 (7.79, 16.65)	0.455
LC, *10^9/L, M (IQR)	1.175 (0.72, 1.75)	1.48 (0.98, 1.87)	0.284
CRP, mg/L, M (IQR)	71.75 (37.65, 225.1)	95.3 (42.675, 172.2)	0.886
Ferroprotein, ug/L, M (IQR)	1782.2 (767.95, 7462.3)	1221.9 (618.6, 2303.05)	0.214
PCT, ng/mL, M (IQR)	0.97 (0.15, 8.56)	0.17 (0.08, 0.87)	0.021
ALT, U/L, M (IQR)	47 (23.75, 76)	43 (21.25, 73.75)	0.976
AST, U/L, M (IQR)	91.5 (38.25, 221.25)	43 (26, 68.75)	0.025
ALB, g/L, M (IQR)	32 (29.55, 34.55)	35.7 (30.7, 39.3)	0.277
LDH, U/L, M (IQR)	347 (248, 405)	216.5 (177, 272.5)	0.066
CR, umol/L, M (IQR)	124.5 (82.5, 214.5)	87 (66.5, 114)	0.029
UREA, mmol/L, M (IQR)	17.9 (5.73, 28.5)	8.55 (5.7, 14.33)	0.06
cTnI, pg/mL, M (IQR)	287 (8.7, 803.4)	15.9 (6.4, 139)	0.189
D2D, ug/mL FEU, M (IQR)	7.51 (1.85, 21)	5.24 (1.39, 18.94)	0.479
FT3, pmol/L, M (IQR)	2.36 (1.8, 2.795)	2.69 (2.3625, 2.91)	0.027
FT4, pmol/L, M (IQR)	10.55 (9.01, 11.69)	16.75 (14.38, 18.46)	/
TSH, uIU/mL, M (IQR)	1.82 (0.62, 2.15)	0.897 (0.41, 1.79)	0.235

Significant differences were observed in PCT (*P* = 0.021), AST (*P* = 0.025) and Cr (*P* = 0.029) levels between the low FT4 group and the normal-high FT4 group. However, no significant differences were found in WBC counts (*P* = 0.455), LC (*P* = 0.284), CRP (*P* = 0.866) and ferroprotein (*P* = 0.214), ALT (*P* = 0.976), ALB (*P* = 0.277), LDH (*P* = 0.066), UREA (P = 0.06), cTnI (*P* = 0.189), and D2D (*P* = 0.479) levels. In addition, relatively lower FT3 levels were detected in the low FT4 group, while no significant difference in TSH levels was observed between the low FT4 and normal-high FT4 groups.

### Association between TSH or FT4 levels and 90-day mortality

**Fig. 1:**
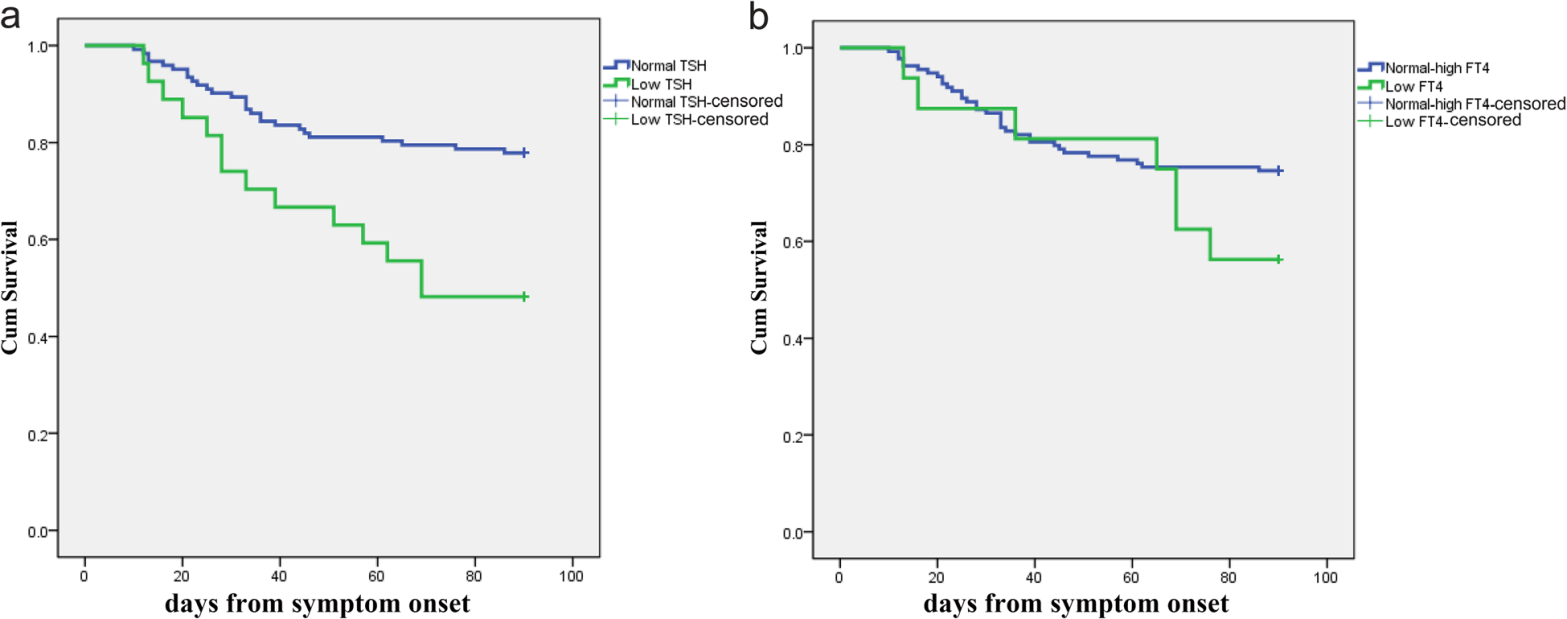
Stratified survival curve for 90-day mortality according to TSH (**a**) or FT4 levels (**b**) in patients with COVID-19 presenting with NTI

The probability of 90-day mortality was assessed using a Kaplan-Meier analysis (Fig. 1). The mortality rate was significantly different between the low TSH group and the normal TSH group (log-rank *P* = 0.001), but it was not significantly different between the low FT4 group and the normal-high FT4 group (log-rank *P* = 0.174).

The Cox proportional hazards model (Table 4) suggested that low TSH levels were independently related to 90-day mortality (hazard ratio = 2.78, 95% CI: 1.42–5.552, *P* = 0.003). Low TSH levels were an independent risk factor for mortality in patients with both COVID-19 and NTI.

**Table 4:** Association of low TSH and mortality in the cox regression analysis model

	Beta	SE	Wald	*P* value	Hazard ratio	95.0% CI	
						Lower	Upper
TSH	1.010	0.342	8.738	0.003	2.745	1.405	5.363
gender	0.174	0.325	0.288	0.591	1.190	0.630	2.250
age	0.619	0.347	3.185	0.074	1.858	0.941	3.668
CRP	1.308	0.736	3.155	0.076	3.699	0.873	15.665
FT4	0.710	0.420	2.859	0.091	2.034	0.893	4.635

## Discussion

NTI refers to alterations in a thyroid test without thyroid lesions in individuals with acute or chronic non-thyroid diseases [6], and it has been reported in many diseases, such as fractures, major trauma, cardiac surgery, meningococcal septic shock, community-acquired pneumonia, kidney disease, lymphocytic leukemia, and diffuse large B cell lymphoma [3]. NTI is characterized by decreased T3 levels and no elevation in TSH levels, always with increased rT3 levels [3]. High, normal or low T4 levels, as well as low or normal TSH levels, have been observed in patients with NTI [3], but the corresponding proportions were unclear. In this study, most the included patients with COVID-19 presenting low FT3 levels had normal FT4 and TSH levels, and then 14% had normal FT4 levels and decreased TSH levels. Although the lung is the key lesioned organ, angiotensin-converting enzyme 2 (ACE2), the receptor for SARS-CoV-2 to invade humans, is expressed at a high level in the thyroid [11, 12]. Many cases of subacute thyroiditis after SARS-CoV-2 infection have been reported [13, 14]. Presumably, NTI was combined with thyrotoxicosis in some patients with COVID-19, especially in those with low FT3 and elevated FT4 levels.

The prognostic effects of NTI on critically ill patients have been extensively studied and recognized, but associations of altered thyroid tests and mortality in patients with NTI are contradictory. In a study focusing on patients with sepsis presenting with NTI, a worse prognosis was observed for patients with combined low T3 and T4 levels compared with those with low T3 levels alone [8]. In a study from Turkey, low FT3 levels and increased FT4 levels were found to be independent predictors of long-term mortality risks in chronic patients with NTI, and TSH levels were not significantly associated with mortality [9]. Another study in Germany found that high TSH, T3 and T4 levels were related to a better prognosis in patients with acute liver failure [15].

For patients with COVID-19, NTI has been found to be significantly associated with the disease condition [1]. In a study conducted in Italy, Ilaria Muller’s team compared patients with and without COVID-19 in a high ICU, and no significant difference was observed in FT3 levels, indicating that the NTI rate might not be increased for patients with COVID-19 treated in the ICU [16]. Lower TSH levels and higher FT4 concentrations were also detected in patients with COVID-19 admitted to the high ICU compared with patients with COVID-19 admitted to the low ICU [16]. The study by Min Chen et al analysed thyroid function in patients with COVID-19. Lower TSH and FT3 concentrations were shown to be associated with the severity of COVID-19 (*p* < 0.001), and TSH levels were significantly lower in severely and critically ill patients with COVID-19 than in non-COVID-19 control patients [7]. In the study conducted by Lania et al., the mortality was higher and the duration of hospitalization was longer in thyrotoxic in-hospital patients with COVID-19 as compared to those with normal thyroid function [17]. Here, mortality and critical illness rates were significantly higher in the low TSH group than in the normal TSH group, and low TSH levels were independently related to 90-day mortality (hazard ratio = 2.78, *P* = 0.003). In addition, the mortality rate was significantly higher in the low FT4 group, but no significant difference in the critical illness rate was found. Although thyrotoxicosis might occur in patients with COVID-19, low FT3-FT4 levels or low FT3-TSH levels suggested a worse prognosis. Critically, severely and mildly ill patients with COVID-19 complicated with NTI were all included in this study, and the analysis of the effects of TSH and FT4 levels on the prognosis was based on low FT3 levels, which partially explained the difference in our results from other findings.

What is the mechanism of low-TSH in patients with COVID-19 and NTI? Why low FT3-TSH level might affect the mortality in patients with COVID-19? Significant differences were observed in the levels of nonspecific inflammatory parameters between the low TSH and normal TSH groups, including WBC counts and CRP and ferroprotein levels. Presumably, inflammation suppresses the hypothalamic-pituitary-thyroid axis [18], which might be one cause of low TSH levels in patients with COVID-19 presenting with NTI [19]. Researchers have not clearly determined whether SARS-CoV-2 invades the pituitary gland and damages TSH-secreting cells [7]. In addition, significant differences in AST, LDH, and UREA levels were observed between the low TSH and normal TSH groups of patients with both COVID-19 and NTI, and prospective clinical and basic studies are needed to explore whether these alterations are the cause of low TSH levels associated with NTI in patients with COVID-19. The deteriorative feedback regulation of pituitary-thyroid axis and the concomitant injuries of other organs may partially explain the mortality, but the pathophysiological mechanism needs further study.

This retrospective study has several limitations. Examinations of rT3 levels, ultrasound inspections of the thyroid and specific antibody tests were not available. In addition, information about steroid and dopamine therapy was unaccessible before hospitalization, and medication data were not collected and analysed. Further studies are needed to determine whether thyroxine supplementation is beneficial based on alterations in thyroid tests.

## Conclusions

Low FT4 and TSH concentrations were associated with mortality in patients with both COVID-19 and NTI; moreover, low TSH levels were an independent risk factor for mortality in these patients.

## Data Availability

Data were available through contacting the corresponding authors.

## Abbreviations

NTI: non-thyroidal illness syndrome
FT3: free triiodothyronine
FT4: free thyroxine
TSH: thyroid stimulating hormone
ICU: intensive care units
WBC: white blood cell
LC: leukomonocyte
CRP: ultrasensitive C-reactive protein
PCT: procalcitonin
ALT: alanine aminotransferase
AST: aspertate aminotransferase
ALB: albumin
LDH: lactate dehydrogenase
cTnI: cardiac troponin I
Cr: Creatinine
UREA: urea nitrogen
D2D: D dimmers
ACE2: Angiotensin-converting enzyme 2
